# Random forests for feature selection in QSPR Models - an application for predicting standard enthalpy of formation of hydrocarbons

**DOI:** 10.1186/1758-2946-5-9

**Published:** 2013-02-11

**Authors:** Ana L Teixeira, João P Leal, Andre O Falcao

**Affiliations:** 1 LaSIGE, Departamento de Informática, Faculdade de Ciências, Universidade de Lisboa, 1749-016, Lisboa, Portugal; 2 Centro de Química e Bioquímica, Faculdade de Ciências, Universidade de Lisboa, 1749-016, Lisboa, Portugal; 3 Unidade de Ciências Químicas e Radiofarmacêuticas, Instituto Tecnológico e Nuclear, Instituto Superior Técnico, Universidade Técnica de Lisboa, 2686-953, Sacavém, Portugal

**Keywords:** Feature selection, Variable importance, High dimensional data, Random forests, Data-mining, Property prediction, QSPR, Hybrid methodology

## Abstract

**Background:**

One of the main topics in the development of quantitative structure-property relationship (QSPR) predictive models is the identification of the subset of variables that represent the structure of a molecule and which are predictors for a given property. There are several automated feature selection methods, ranging from backward, forward or stepwise procedures, to further elaborated methodologies such as evolutionary programming. The problem lies in selecting the minimum subset of descriptors that can predict a certain property with a good performance, computationally efficient and in a more robust way, since the presence of irrelevant or redundant features can cause poor generalization capacity. In this paper an alternative selection method, based on Random Forests to determine the variable importance is proposed in the context of QSPR regression problems, with an application to a manually curated dataset for predicting standard enthalpy of formation. The subsequent predictive models are trained with support vector machines introducing the variables sequentially from a ranked list based on the variable importance.

**Results:**

The model generalizes well even with a high dimensional dataset and in the presence of highly correlated variables. The feature selection step was shown to yield lower prediction errors with RMSE values 23% lower than without feature selection, albeit using only 6% of the total number of variables (89 from the original 1485). The proposed approach further compared favourably with other feature selection methods and dimension reduction of the feature space. The predictive model was selected using a 10-fold cross validation procedure and, after selection, it was validated with an independent set to assess its performance when applied to new data and the results were similar to the ones obtained for the training set, supporting the robustness of the proposed approach.

**Conclusions:**

The proposed methodology seemingly improves the prediction performance of standard enthalpy of formation of hydrocarbons using a limited set of molecular descriptors, providing faster and more cost-effective calculation of descriptors by reducing their numbers, and providing a better understanding of the underlying relationship between the molecular structure represented by descriptors and the property of interest.

## Background

An area where data-mining techniques are increasingly playing an important role is chemoinformatics, considering that the number of known and synthesized chemical compounds is growing exponentially, but the determination of their properties as well as biological activities is a time consuming and costly process and is lagging severely behind [1,2]. These complex non-homogeneous data lead to the development and application of data-mining tools to extract and understand the underlying quantitative structure-property/activity relationship (QSPR/QSAR) [3-5]. QSPR/QSAR methods are widely used for prediction and their goal is to relate molecular descriptors, from molecular structure, with experimental chemical, physical and/or biological properties by means of data-mining methods [6-10]. The three major difficulties in the development of QSPR/QSAR models are (1) quantifying the inherently abstract molecular structure, (2) determining which structural features most influence the given property (representation problem) and (3) establishing the functional relationship that best describes the relationship between these structure descriptors and the property/activity data (mapping problem) [8-12]. The first difficulty can be overcome by the use of calculated molecular descriptors, developed to quantify various aspects of molecular structure [13]. In fact, this approach is one of the causes of the second difficulty since thousands of molecular descriptors are currently extant [13,14]. The problem lies then in the identification of the appropriate set of descriptors that allow the desired property of the compound to be adequately predicted. To accomplish this and to find the optimal subset of descriptors that describes the relationship between the structure and the property/activity data, several statistical and data-mining methods are commonly used for feature reduction and selection [15,16]. Frequently, it has been observed that certain descriptors appear to be relevant for a specific problem (for example, the molecular weight of a drug is an important parameter that may affect the capacity of a drug to permeate across the blood-brain barrier [17]). However, in general, this task cannot be completely achieved manually, given the complex non-linear nature of the structure-property/activity relationships and the high number of existing molecular descriptors. An optimal solution for this problem requires an exhaustive search over all possible subsets. Considering the high number of molecular descriptors (*n*) and the limited knowledge on the amount of necessary descriptors (*p*), it is required to try for each *p* the sum of the *n*th row of the binomial coefficients, which involves 2^*n*^ possible combinations. This exhaustive enumeration of each subset is computationally impractical, except for small problems. Therefore, a reasonable alternative is then the use of an heuristic approximation that may be able to find the best possible subset of descriptors within the available computational resources [18].

Several studies have investigated approaches to solve the descriptor selection problem in QSPR/QSAR [19-21]. Any set of descriptors may be used in a QSPR/QSAR model and therefore techniques to reduce the dimensionality or select the best combination of descriptors are very important [21]. The first group of techniques, feature reduction, aims to map the original high-dimensional data into a lower-dimensional space obtaining transformed features (generally linear combinations of the original features) [21]. The construction of models based on feature reduction such as principal component analysis (PCA) [22] and partial least squares regression (PLS) [23] compress the original dataset generating a smaller number of variables. PCA transforms the original dataset into orthogonal components, constructed by linear combinations of the existing variables. These are arranged in descending order according to the percentage of variance each component explains. Therefore the first components (principal components) are expected to translate the main sources of variability of the data, and may be better suited for modelling purposes [21]. However, PCA does not reduce the number of features needed for prediction, it only reduces the number of parameters in the model, as all features may be present in each component. The second group of techniques, feature selection, aims to choose an optimal subset of features according to an objective function [21,24]. The feature selection can be: (1) objective if it uses only molecular descriptors (independent variables), removing redundancy amongst all the descriptors using the correlation matrix or (2) subjective if it also uses the property of interest (dependent variable) to identify the subset of descriptors that best map a relationship between structure and property [25]. For that purpose several search algorithms have been devised, ranging from simple heuristic approaches [26,27] which perform a "greedy" search of the best subsets of variables such as forward selection, backward elimination or stepwise procedures to further elaborate methodologies including simulated annealing [28] and evolutionary programming [29] such as genetic algorithms [30]. These methods allow a stochastic evolutionary search of the possible solution space of a problem aiming for the selection of an optimal non-redundant set of variables, if sufficient computational resources are provided [21]. Other recent articles present multi-phase methodologies, in which the subsets of descriptors are selected and assessed using different algorithms [31]. The problem lies in selecting the minimum subset of descriptors that can predict a certain property with a good performance, less computational/time cost and in a more robust way, since the presence of irrelevant or redundant features can cause a poor generalization capacity.

Due to the high rate of new compounds discovered each day and the fact that laboratory techniques for experimental measurements are still expensive, there is a significant gap between the number of known chemical compounds and the amount of experimental thermochemical property data in the literature. Thus it is clear the great need to foster the application of prediction methods with a good predictive performance when experimental values are not available. It is also important to note that generally in QSPR problems and specifically in the prediction of enthalpy of formation problem, small improvements in the prediction capacity are very important, considering that they can result in further improvements in efficiency and safety of chemical processes in the chemical industry.

Some methods have been developed for predicting thermochemical properties of molecules, ranging from group/bond additivity, high-level theoretical calculations and quantitative structure–property relationships (QSPR) methods. The empirical additivity methods are heavily-parameterized schemes for interpolating between experimental values with a different range of applicability, different reliability (precision and accuracy) and usually limited by the high number of parameters which tends to affect the ability to extrapolate to data outside the training set and the existence of parameters that have not been estimated due to the lack of experimental data [32]. The most frequently used group additivity method was proposed in 1958 by Benson and Buss [33]. Another frequently used method to predict thermochemical properties is based on bond additivity and it was proposed by Laidler in 1956 [34]. Laidler’s bond additivity method has been refined and extended by other authors (*e.g.*[35] and [36]). High-level theoretical calculations can be applied to estimate thermochemical properties for small to medium sized molecules. These kinds of methods obtain molecular properties from the most fundamental level of molecular information: electronic (such as number of electrons) and spatial molecular structure (such as location of the nuclei) [37]. However, high-level theoretical calculations are very intensive computationally and require a substantial time investment, limiting their application to small/medium size molecules. An additional alternative for modelling the physical-chemical properties is to resort to the structure of the molecule through the quantitative structure–property relationships (QSPR), which also have proved to be useful in this respect. In the specific case of prediction of enthalpies of formation of specific classes of compounds, some QSPR models have been used such as the ones developed by Mercader *et al*[38] which predicts enthalpy of formation of hydrocarbons based on a specific class of molecular descriptors, Ivanciuc *et al*[39] which predicts enthalpy of formation of alkanes at 300 K based on 3 atomic structural descriptors derived from the molecular graph investigated one at a time**,** Yu *et al*[40] which predicts enthalpy of formation of alkyl derivatives based on a topological index, Yao *et al*[41] which predicts enthalpy of formation of alkanes (between C_6_ and C_10_) at 300K based on radial basis function neural networks using 35 structural/topological calculated descriptors that were reduced to four principal components and Vatani *et al*[42] which predicts enthalpy of formation at standard state of different types of compounds based on a multivariate linear genetic algorithm using 5 structural descriptors calculated and selected from a pool of 1664 descriptors.

In this manuscript, we present an alternative approach to select molecular descriptors inspired by a methodology proposed by Genuer *et al*[43] and applied to prediction of standard molar enthalpy of formation of gas phase at 298.15 K for hydrocarbon compounds. Genuer *et al*[43] proposes a two-steps procedure: (1) preliminary elimination and ranking, sorting the variables in decreasing order of standard deviation of Random Forests scores of importance from a series of runs and elimination of variables with small importance; (2) variable selection for prediction, starting from the ordered variables by constructing an ascending sequence of Random Forest models, testing the variables stepwise and retaining it only if the error gain exceeds a certain threshold. The algorithm Random Forest is widely used in the prediction context (classification and regression) given that it has several features that make it suitable for a QSAR/QSPR dataset [44-46]. These include good predictive performance even when there are more variables than observations, capacity to handle a mixture of categorical and continuous descriptors, measures of descriptor importance and due to its nature encompassing a large number of simple models, it largely reduces the problems caused by over fitting [44-46]. However, there are few works in the literature using Random Forests in the context of descriptor selection. To the best of our knowledge, beyond the work of Genuer *et al*[43] , there is another study in the literature that uses random forests for gene selection in classification problems [47], for that purpose several forests are generated iteratively and at each iteration the variables with the smallest variable importance are discarded; the selected set of variables is the one that yields the smallest prediction error. In this manuscript we propose a hybrid approach that also uses Random Forests, but differently from Genuer *et al*[43], using the quantification of the average variable importance from a series of runs provided by this method, as a tool for molecular descriptors selection. This ranking can be used to build a predictive model, without eliminating any variables, using any other machine learning prediction method, in this case and differently from Genuer *et al*[43], Support Vector Machines [48], inserting the variables stepwise in order to find a good balance between the number of variables and prediction error.

The two main objectives of this hybrid methodology are: (1) obtain a set of descriptors that are most related to the property of interest using the variable importance index calculated by random forests and (2) obtain the smallest possible set of molecular descriptors that can still achieve a good predictive performance that generalizes well even if the ratio between the number of variables and number of observations becomes unfavourable. In order to assess results, and have a reference of the developed models performance, the results will be compared with the ones obtained for models without a feature selection step and for models using other feature selection/reduction techniques such as Principal Components Analysis and Genetic Algorithms. Finally, the model performance will be tested using an independent validation set.

## Results

### Prediction models

To verify the importance of feature selection methods for the prediction of standard enthalpy of formation of gas phase of hydrocarbons the following methodology was envisaged: in the first place it is necessary to assess model behavior without any feature selection. Secondly, three variable reduction strategies were tested, that include the use of i) support vector machines (SVMs) with principal components analysis for all the feature set space; ii) using genetic algorithms coupled with SVMs for feature selection; iii) use the ranked features list as produced by random forests for searching a minimal feature set to train a SVM model.

#### Model development without a feature selection/reduction step

In order to confirm that it is possible to eliminate variables which are not informative as predictors of the property of interest, the first step is to present model results with the whole set of molecular descriptors (1485). For that purpose both Support Vector Machines (SVMs) and Random Forests (RFs) were tested.

Random Forests have two model parameters that condition the model results, namely, the number of variables randomly sampled at each node to be considered for splitting and the number of trees in the forest. A preliminary systematic evaluation of both parameters on the training set led us to conclude that 240 variables at each node and 500 trees in the forest should be used. Larger values than these did not yield better results. For each parameter combination, the full dataset of 364 molecules was used within an out-of-the bag cross validation procedure, as is usual for random forest models. The best model reached a root mean square error (RMSE) of 50.28 which corresponds to a cross-validated proportion of variation explained (q^2^) of 0.9393 (Table 1). Using Epilson-SVM with a preliminary tuning of the radial basis function (RBF) kernel parameters (which included the cost parameter that controls the trade off between allowing training errors and forcing rigid margins with the value 100 and the gamma parameter that controls the shape of the separating hyper plane with values ranging from 1 × 10^-3^ to 1 × 10^-6^ depending on the number and nature of descriptors used) the obtained RMSE of the 10-fold cross validation was 44.47, corresponding to a q^2^ of 0.9520 (Table 1).

**Table 1:** Summary of the results (10-fold cross validation) obtained for all the models

**Feature selection**	**Feature selection technique**	**Number of variables/PC**	**Machine learning model**	**RMSE**^**¤**^	**q**^2^_cv_^§^
No		1485	RF^‡^	50.28	0.9303
		1485	SVM^†^	44.47	0.9520
Yes	PCA^*^	28 PC^#^	SVM^†^	34.87	0.9671
	GA°	58	SVM^†^	47.1	0.9391
	RF - VI^ǂ^	89	SVM^†^	34.1	0.9686

^¤^Root Mean Square Error; ^§^cross validated squared correlation coefficient; ^‡^Random Forests; ^†^Support Vector Machines; ^*^Principal Components Analysis; ^#^ Principal Components; ° Genetic Algorithms; ^ǂ^Variable Importance using Random Forests.

#### Model development with a feature selection/reduction step

Principal components analysis to reduce the number of molecular descriptors

**Figure 1:**
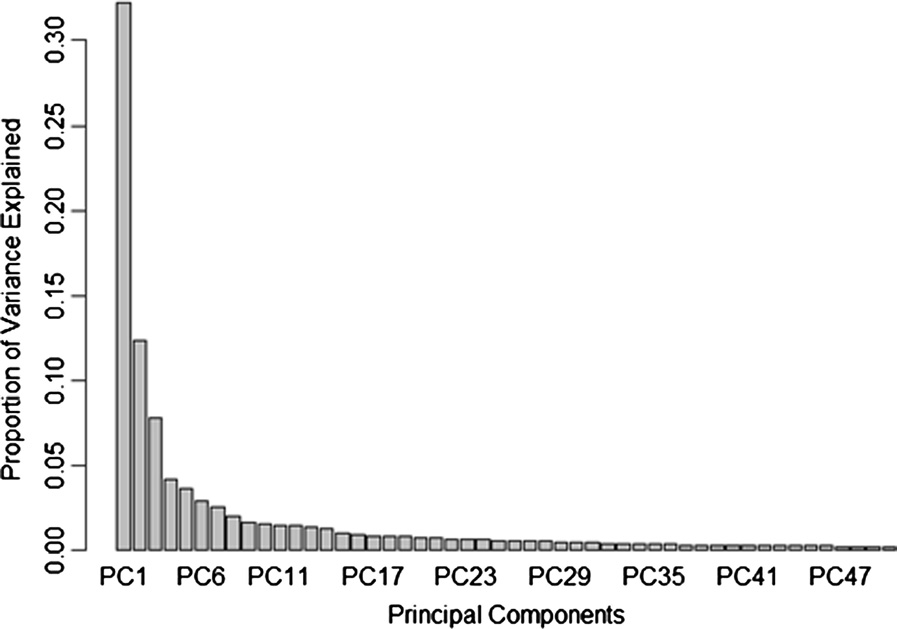
Proportion of variance in the descriptor set that is explained by each principal component (for readability the plot was truncated after the fiftieth variable).

Analyzing the correlation matrix between all the variables in the dataset in study, it is possible to verify that the variable space presents a significant degree of redundancy. In order to test how the correlation between the variables affects the model performance we will use Principal Component Analysis (PCA) to remove linear correlations and compare the results. To ensure adequate comparison of the values for each variable, each one was centered and scaled to mean equal to zero and standard deviation equal to 1.0. The plot represented in Figure 1 shows the proportion of variance in the dataset that is explained by each principal component (PC). The 3 first PCs are enough to explain 52.4% of the variance in the original dataset and the most significant 123 principal components are sufficient to explain 99% of the variance in the original dataset (*Additional file*1).

**Figure 2:**
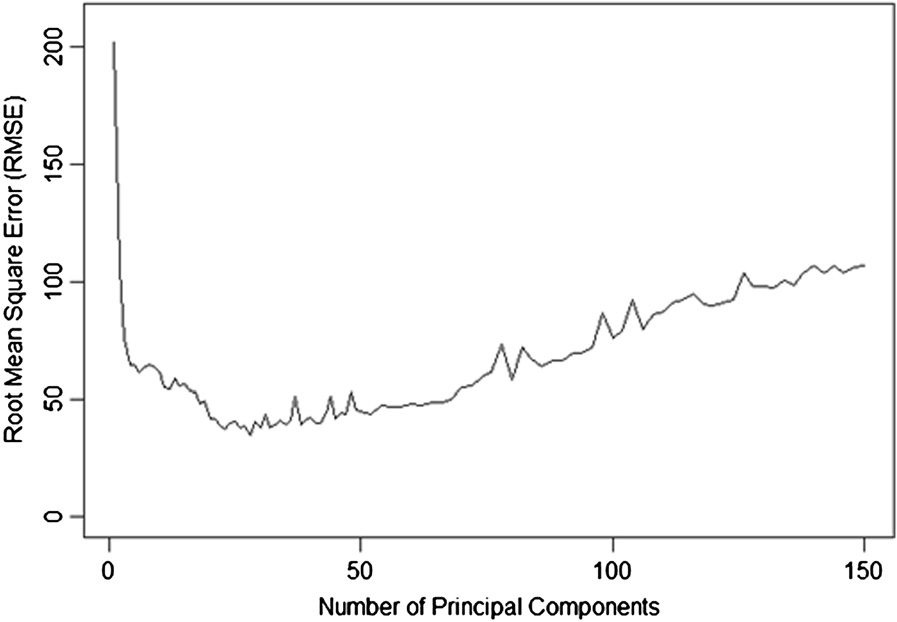
Comparison of the root mean square error (RMSE) for each predictive SVM model using an increasing number of principal components in descending order of proportion of variance explained (previously determined using principal components analysis).

To use PCs as model inputs, the same question of how many components are necessary for adequate modelling is pertinent. Therefore, a stepwise approach for model construction was followed. Accordingly, several SVM models were fitted adding progressively more components following the decreasing order of the proportion of variance explained, until 150 components were present. Each model was evaluated using a10-fold cross validation. It was verified that the best model, providing the minimum RMSE (34.87), was obtained using the first 28 PCs (Table 1), and from this point on the prediction performance decreases for each PC added (Figure 2).

Genetic algorithms for feature selection

A genetic algorithm procedure for variable selection was adapted to this problem and implemented. The algorithm parameters were subjected to preliminary screening in order to ensure that the heuristic is able to adequately search the variables’ solution space, evaluating each set of variables found during the process with a SVM, and using the cross-validated score to rank and select each proposed subset of variables. The GA strategy that produced the best results was by using a population of 80 chromosomes, with a mutation rate of 2.5%, and cross over was verified as irrelevant. Initial solutions used an initial density of 4.0% meaning that, at most, 59 features are being selected for each model. During the optimization process it was verified that there were no improvements in the model performance after 1000 generations. The genetic algorithm heuristic was repeated 10 times and the final result is the average of the best solution in each run [49]. The obtained RMSE value was 47.10, corresponding to a q^2^ of 0.9391, using an average of 58 variables (Table 1). It is important to note that the list of variables selected with this method varied widely within models, with only 2 or 3 common variables per run, showing that this method although capable of producing solutions of similar quality than using all the variables, is not coherent on the set of features selected (*Additional file*1). However, it is noteworthy that approximately half of the selected descriptors are Daylight fingerprints [50], representing certain structural fragments.

Variable importance index from Random Forests

**Figure 3:**
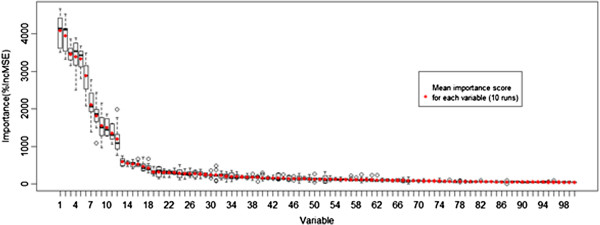
Boxplots depicting the distribution of the importance score (percentage of prediction error increased when the variable is permuted randomly) of each variable, ordered by mean importance (marked with red dots) resulting from 10 runs of the model.

In order to find the ordered list of variables according to their importance, the random forest model fitted previously was used and the importance of each variable in the final model was recorded. Due to the stochastic nature of the random forest approach, this procedure was repeated 10 times, and in the end this rank order was averaged for each variable. The variables were then sorted according to the average variable importance in descending order (Figure 3). These results clearly suggest that there are six very important descriptors and six moderately important ones while the others are of small importance and that the group of most important variables is not interchangeable since they have a clear difference in the quantity increased in prediction error. The results appear to be coherent and robust, with the first 20 descriptors occupying coherently the first positions in the rank, clearly illustrating the importance of each in the current problem (*Additional file*1).

**Figure 4:**
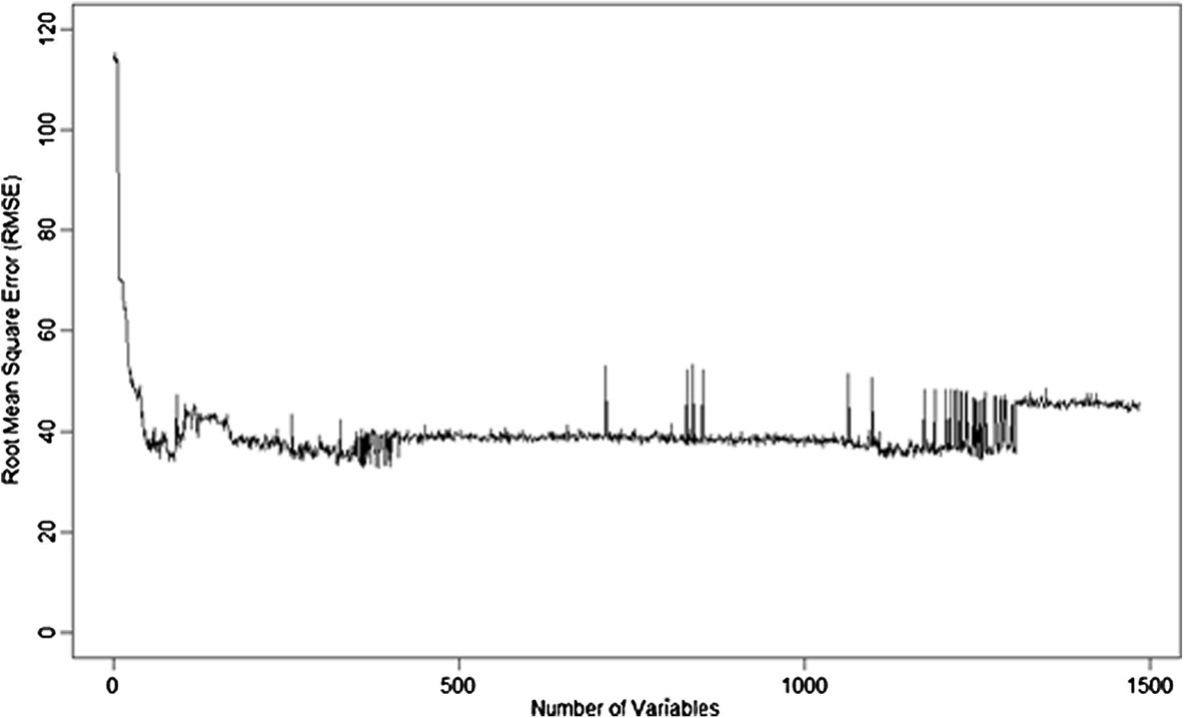
Comparison of the root mean square error (RMSE) for each predictive SVM model using an increasing number of variables in descending order of importance (previously determined using random forests).

With the produced descriptor rankings, the procedure followed was similar to the one used for PCA where each variable was introduced stepwise into a new model fitted with SVMs, and recording the statistical results for each new feature added. The 10-fold cross validation results for each iteration are shown in Figure 4 and its analysis show that a minimum RMSE (32.82) corresponding to a q^2^ of 0.9706 was reached when 385 variables were used. However it can be verified that the number of variables can be reduced to 89 without losing much predictive power, with an RMSE of 34.10 and a q^2^ of 0.9686 (Table 1). Nonetheless, it can be verified that, in general, the predictive power of the models does not increase after 200 variables.

Table 1 summarizes the results obtained for the different approaches presented above, comparing the performance of the models using or not a feature selection/reduction step.

Random forests are robust techniques, however due to the orthogonal division of the space their predictive performance (RMSE = 50.28) is not as good as the one obtained with SVMs (RMSE = 44,47) even in the absence of the feature selection step. SVM models, on the other hand, are sensitive to the number of input variables, and using a smaller descriptor set have, in general, better predictive power than larger descriptor sets. The use of genetic algorithms has produced descriptor sets that are able to produce good results with a limited amount of variables, yet we did not found any coherency in the descriptors selected, which precludes the use of this technique as a reliable tool for selecting variables. PCA has produced model results that are statistically similar to the variable ranking approach as considered by random forests, yet, PCA still requires the computation of all 1485 descriptors for its application which is a relevant shortcoming. The fact that the results produced by PCA and variable ranking approach as considered by random forests are similar is an evidence, as also argued by some authors [51], that the effects of correlation between descriptors mostly affects the interpretation of the model, with only slight effect on its predictive power. Thus the random forest based variable ranking approach is the natural choice for a final model, which, for the present problem, is able to reach robust models using only 89 molecular descriptors.

### Model Validation with an Independent Validation Set

All the results presented so far have been obtained using 10-fold cross validation. It is important nevertheless to use an external and independent validation set to perform an unbiased validation of the selected model [8,10,52]. Therefore to assess the model validity, it was tested with an independent validation set of 100 molecules, which were never considered in any of the training phases. The predictive performance of the 89-features model to this data was similar to the one obtained with 10-fold cross-validation, with an RMSE of 48.64 and a predictive proportion of variation explained (Q^2^) of 0.9607. These values confirm the robustness of the approach and the effectiveness of the feature selection phase in capturing the relevant information for modelling.

## Discussion

### Selected chemical descriptors

Different feature selection/reduction techniques were applied to select the most important descriptors in order to predict the property of interest. The stability of these methods is very important, since ideally, in the same conditions, different runs of each method should not influence the feature subset selection. The most important descriptors selected by the three methods are very different between each other, however the descriptor average molecular weight (AMW) appears as important to both genetic algorithms and variable importance calculated by random forests. Genetic algorithms select mostly Daylight fingerprints, while variable importance calculated by random forests give more importance to the 2D and 3D descriptors calculated by E-DRAGON. In terms of stability, genetic algorithms are not coherent on the set of features selected since, in general, only 2 or 3 variables are common per run while using variable importance calculated by random forests the list of most important descriptor is coherent. It is difficult to assess the relative importance/contribution of each variable in the principal components calculated by principal components analysis.

**Figure 5:**
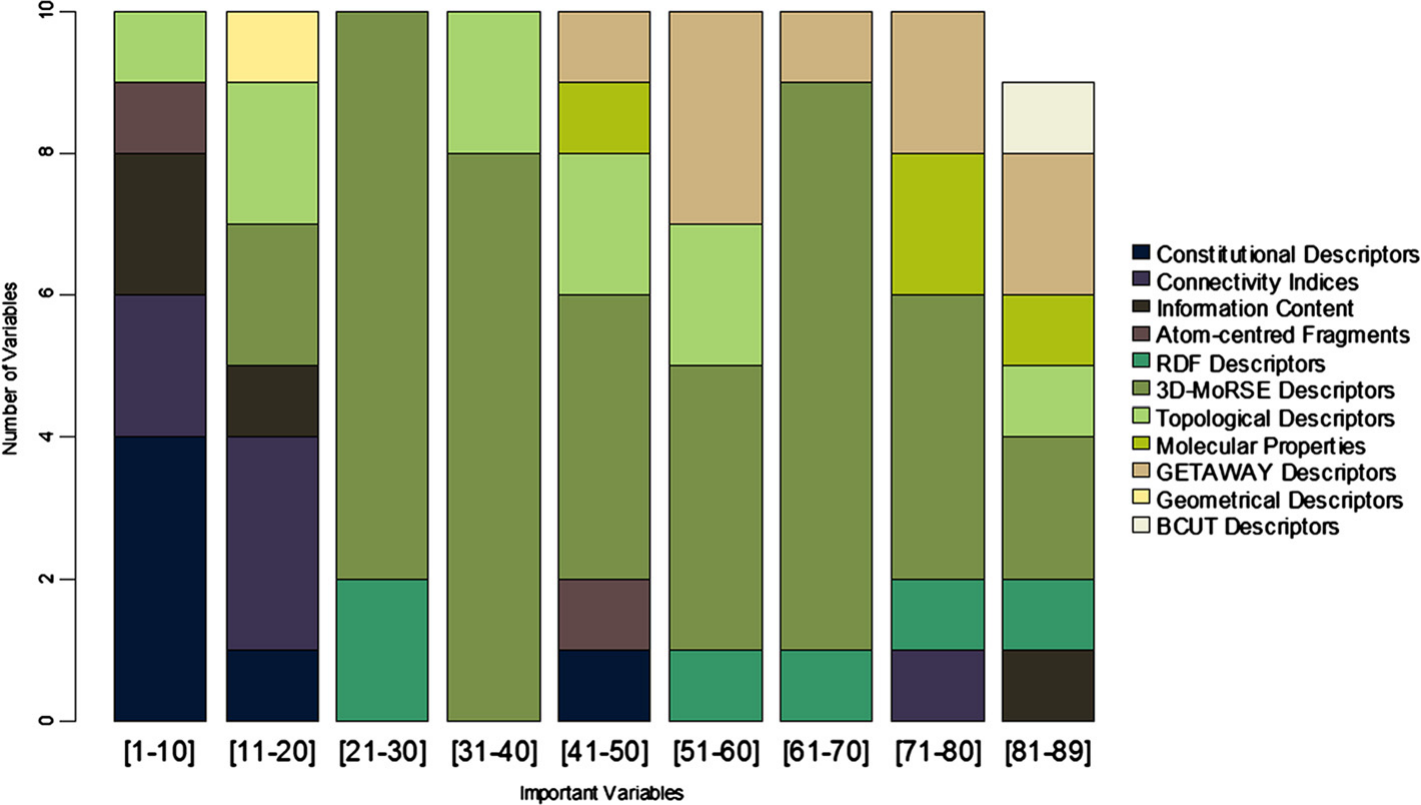
Distribution of the 89 most important variables by classes of descriptors.

The 89 most important descriptors selected using variable importance calculated by random forests were individually analyzed. In a first step these were grouped into general classes (Figure 5). These descriptors are derived from different models and approaches, but they can be loosely grouped according to their information content: a) *Constitutional descriptors*, reflecting the molecular constitution and independent from molecular connectivity and conformations; b) *Connectivity indices and Topological descriptors*, reflecting the topology of a given structure, calculated from the vertex of the atoms in the H-depleted molecular graph; c) *Information content indices*, reflecting the neighborhood of an atom and edge multiplicity; d) *BCUT descriptors*, reflecting atomic properties relevant to intermolecular interactions, calculated from the eigenvalues of the adjacency matrix; e) *Atom-centred fragments*, reflecting the presence of a set of defined structural fragments; f) *Radial Distribution Function (RDF) descriptors*, reflecting the molecular conformation/geometry in 3D, based on the distance distribution in the molecule; g) *3D-Molecule Representation of Structures based on Electron diffraction (MoRSE) descriptors*, reflecting 3D information based on the 3D coordinates of the atoms by using the same transformation as in electron diffraction; h) *GEometry, Topology and Atom-Weights AssemblY (GETAWAY) descriptors*, reflecting the 3D molecular geometry provided by the leverage matrix of the atomic coordinates; i) *Geometrical descriptors*, reflecting the conformation of a molecule based on their geometry; j) *Molecular Properties*, calculated using models or semi empirical descriptors [14]. A list of the 89 descriptors in decreasing order of variable importance is provided in the *Additional file*2.

Although the 10 most important variables reflect mainly 2D information (constitutional, connectivity, information content and atom-centred fragments descriptors), the most common type of descriptors, with 40 variables, reflects 3D information (3D-MoRSE descriptors). The most important variable found for the prediction of the standard enthalpy of formation of gas phase is the average molecular weight, which represents the sum of the atomic weights of the atoms in the molecule divided by the number of atoms in the molecule (including hydrogen atoms). Unlike the molecular weight, this descriptor does not give an idea of the size of the molecule, but about the branching, type of atoms and bonds and therefore it has a good capacity to distinguish different families of hydrocarbons. Contrasting to the sets of variables selected by the model trained with genetic algorithms, which have a high accounting for fingerprints, this set of variables does not contain fingerprints.

### Prediction errors analysis

**Figure 6:**
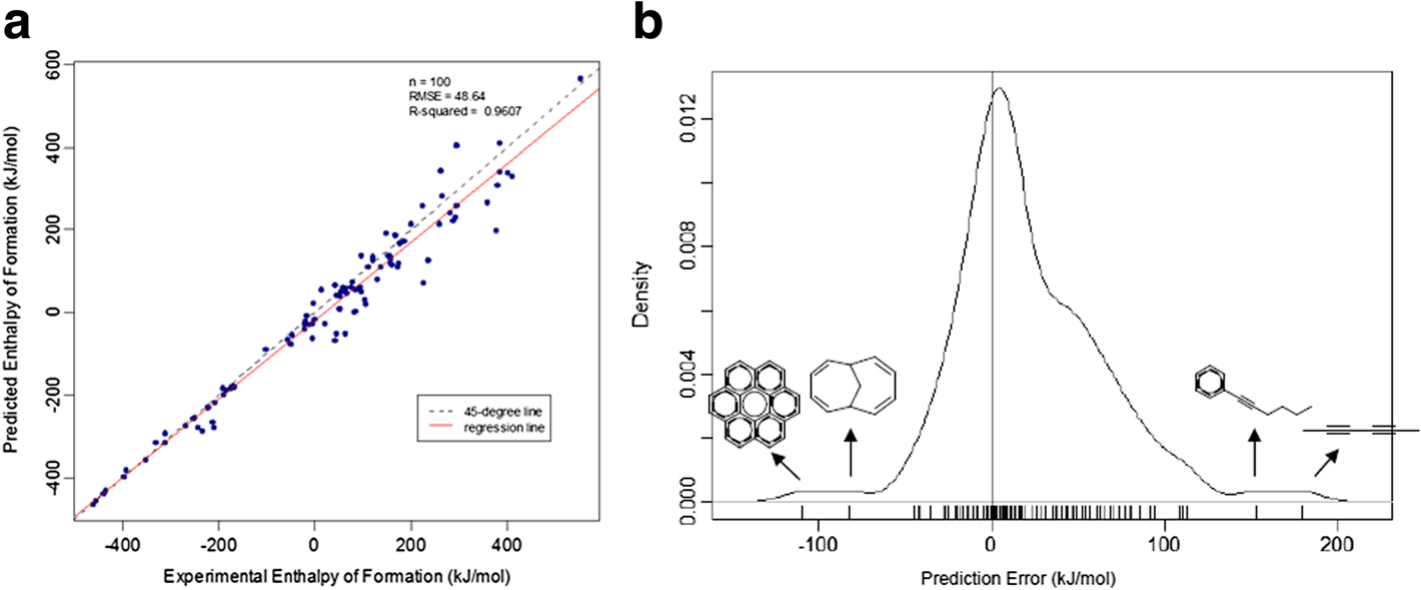
**a) Plot of experimental versus predicted values of enthalpy of formation of gas phase (kJ/mol) using the independent validation set. b) Density plot of the differences between the observed values and the predicted values using the independent validation set. **The structure of the compounds with most extreme prediction errors are indicated, the positive errors correspond to compounds with triple bonds (hexa-2,4-diyne and hex-1-ynylbenzene) and the negative errors correspond to compounds with more than one cycle (coronene and bicyclo[4.4.1]undeca-2,4,7,9-tetraene).

The experimental values of enthalpy of formation of gas phase (kJ/mol) were compared to the predicted values using the independent validation set and represented in a scatter plot, with an RMSE of 48.64 and a Q^2^ of 0.9607 (Figure 6a). The majority of the data points are concentrated around the line of equality between the experimental and predicted value of the property (45-degree line) therefore, the relationship between them is strong. The distance of each symbol from the 45-degree line corresponds to its deviation from the related experimental value. The regression line indicates that generally the model predicts values close to the equality with a small deviation showing that the model is predicting with smaller values than the observed ones. The prediction errors obtained for the independent validation set were then further analyzed and are represented in the Figure 6 - b). Similarly to what has already been observed, the model is predicting the enthalpy of formation with a left bias (smaller values than expected) and the most probable error is 4.10. The compounds with higher errors are the alkynes, probably due to the fact that this type of compounds are over-represented in the validation set with 12 compounds while only 4 alkynes exist in the training set and the latter is more than 3.5 times larger than the former. Therefore, this under-representation may be affecting the selection of descriptors to represent this type of compounds and their relationship with the property of interest. Removing the two alkynes (hexa-2,4-diyne and hex-1-ynylbenzene) with higher prediction errors, the RMSE decreases around 11.6% to 42.99 and a Q^2^ of 0.9684, which is an indicator that these type of compounds are not well represented in the training set. Another class of hydrocarbons with high error rate are the polycyclic compounds, although the experimental confidence on these values is lower than for the rest of the dataset, the fact that they have complex structures and conformations may be the cause for a higher difficulty establishing a relationship between their representation and the property of interest.

## Conclusions

It is unrealistic to think that all descriptors of a molecule contain useful information for a specific modelling problem. It is further acknowledged that models with larger numbers of variables are not necessarily better. Furthermore, smaller models tend to generalize better than larger models, and tend to be more robust statistically. Therefore, after numerical descriptors have been calculated for each compound, its number should be reduced to a set of them that are information rich while being as small as possible. The proposed approach uses random forests, not as modelling tools for themselves, but as a method capable of identifying the most important features of a given modelling problem, which are then used as input variables to SVM models. It is important to note that random forests were the selected algorithm due to the enumerated advantages; however, in principle, any machine learning able to produce a ranking of variable importance could be applied. The second part of this hybrid algorithm uses a ranked list of the variables, ranging from the most to the least important, to train SVM models using a stepwise approach of adding one variable for each model according to its predefined rank. Once again it is important to note that, in principle, any non-linear machine learning method could be applied. The parameters of both models were optimized and the effect of correlated variables studied. From the analysis of the obtained results for a manually curated QSPR dataset, we can conclude that the presented methodology performs well for high-dimensional data and it is robust even in the presence of highly correlated variables. The feature selection step yields lower prediction errors (RMSE = 34.10) with a small number of variables (89). When comparing it to using the model with all the available descriptors (1485), the current 89-variable model was able to produce models with an RMSE 23% lower. These reduced errors are relevant in thermochemistry with significant chemical and economical importance. It is then safe to conclude that SVMs alone are not able to perform a good optimization, and by combining with a variable selection step we can obtain a minimum subset of important variables to train a faster and more robust model, yielding better prediction performance.

The predictive model was validated with an independent set to assess its performance in new data and the results were similar to the ones obtained for the training set with 10-fold cross validation.

The purpose of the current work was to suggest and apply a methodology able to reduce the variable space while preserving (even increasing) the model prediction capabilities, thus reducing the redundancy and correlation between variables. The final suggested model used only 6% of the full set of descriptors and produced better results than a model using all of them. Nonetheless, the full model uses 89 variables, and we cannot exclude the possibility of variable correlation and/or overfiting. Yet, the use of cross validation throughout the full model selection process coupled with a very stringent model evaluation with an independent data set with data from different sources, which produced similar results to the training-validation dataset, is a guarantee that these problems are minimized and of reduced impact respective to its application to a real world scenario.

In summary, the proposed methodology improves the prediction performance of standard enthalpy of formation of hydrocarbons using as molecular representation a set of molecular descriptors, providing faster and more cost-effective calculation of descriptors by reducing their number, and providing a better understanding of the underlying relationship between the molecular structure represented by descriptors and the property of interest.

### Data and methods

The process of model development in QSPR is generally divided into three steps: data preparation, data analysis, and model validation [8-11]. The first stage includes the collection and cleaning of a dataset for the study and the selection of the best molecular representations [8-10]. The second stage deals with the selection of a statistical multivariate data analysis and correlation techniques [8-10,12]. The third stage validates and evaluates the developed model [10,52]. As the problem discussed in this study is centered on models for feature selection, the second stage was performed several times as the purpose was to iteratively search for the optimal parameters for a model or for establishing the minimal number of variables necessary for adequately fitting a model without losing its predictive power. In order to ensure minimal bias in evaluating our results an exhaustive validation procedure was followed, both for model selection as well as for final model assessment. Therefore, during the model evaluation phase, each model was always internally validated using ten-fold cross validation (for SVMs) or out-of-bag prediction (for Random Forests). After selecting a final model with a predefined set of variables, it was further validated with an external validation set never used on any phase of the training process and descriptor selection, and with a different origin.

For the present section, initially the training set and the independent validation set are described, followed by the main modelling methodologies used, namely support vector machines and random forests. Also described are the procedures used for variable reduction/selection either based on random forests variable ranking, principal components analysis and genetic algorithms.

### Data and data pre-processing

#### Training set

Hydrocarbon compounds consist entirely of hydrogen and carbon. For this reason and because hydrocarbon fragments are found in most types of compounds, a good prediction method should give an accurate and consistent estimation. Considering that the quality and prediction capabilities of any method strongly depend on the amount and quality of the experimental data used for its development, the dataset used to model development was collected and manually curated by chemistry experts and it is available online on the ThermInfo database ( http://www.therminfo.com). The dataset covers different types of hydrocarbons (Table 2) and it contains 364 compounds structurally characterized and with experimental values for the standard molar enthalpy of formation of gas phase at 298.15 K (Δ_f_*H*^0^).

**Table 2:** Distribution of the compounds in the training and independent validation sets into the different types of hydrocarbons

** Type of hydrocarbon**	**Number of compounds in the training set**	**Number of compounds in the independent validation set**
Non-Cyclic	131	35
- Alkanes	66	7
- Alkenes	61	16
- Alkynes	4	12
Cyclic	233	65
- Aromatic	85	19
- Polycyclic	62	15
- Non-Aromatic	148	46
- Polycyclic	67	25
**Hydrocarbons - Total**	**364**	**100**

**Figure 7:**
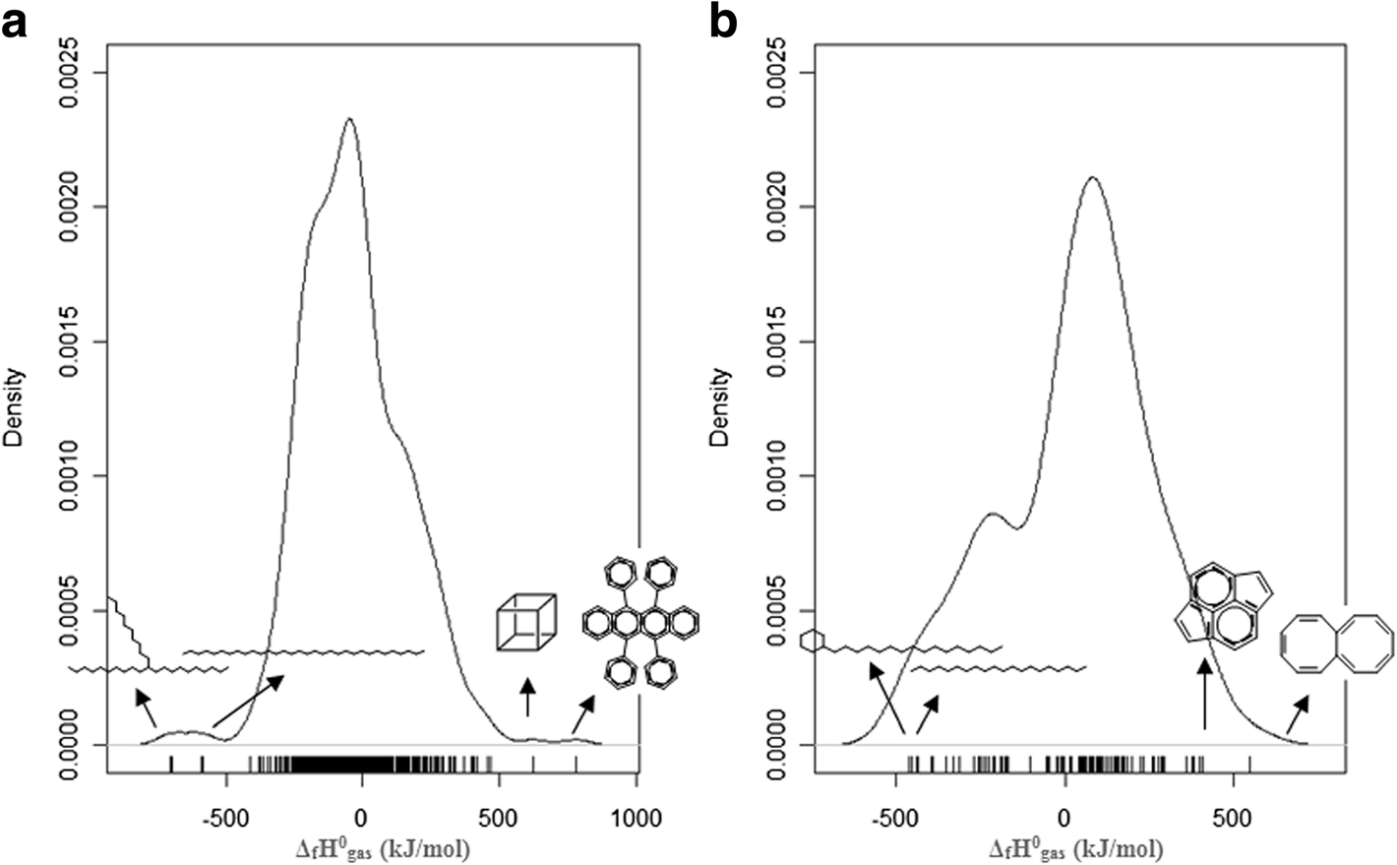
**Density plot showing the distribution and variation of the standard enthalpy of formation of gas phase at 298.15 K in the: a) training set, indicating the structure of the compounds with the most extreme values (maximum: 5,6,11,12-tetraphenylbenzo[b]anthracene and pentacyclo[4.2.0.0**^**2,5**^**.0**^**3,8**^**.0**^**4,7**^**]octane; minimum: dotriacontane and 11-decylheneicosane); b) independent validation set, indicating the structure of the compounds with the most extreme values (maximum: (1Z,3Z,5Z,7Z,9Z,11Z)-octalene and cyclopenta[fg]acenaphthylene; minimum: hexadecylcyclohexane and eicosane).**

The values range from −705.8 kJ/mol to 780.9 kJ/mol, with a mean value of −33.6 kJ/mol and standard deviation of 190.8 kJ/mol. The distribution and variation of the dependent variable is shown in Figure 7a). Although the values have a large range of distribution, the major part of the compounds' enthalpy is located between -500 and 500 kJ/mol. A complete table with ThermInfo ID, CASRN, compound name, SMILES and experimental values for the standard molar enthalpy of formation of gas phase at 298.15 K is provided in the *Additional file*3.

#### Independent validation set

One of the simplest and most widely used measures of generalization is testing the model performance on an independent validation set. The validation set was collected from two different sources, NIST Web book (version 2012) [53] and CRC Handbook of Chemistry and Physics (version 2010) [54]. The validation set covers different molecules that were not part of the training set and it contains 100 compounds structurally characterized and with experimental values for the standard molar enthalpy of formation of gas phase at 298.15 K (Δ_f_*H*^0^) for which the same molecular descriptors used in the training set were calculated (Table 2). The Δ_f_*H*^0^ values range from -460.50 kJ/mol to 551.50 kJ/mol, with a mean value of 30.02 kJ/mol and standard deviation of 221.2 kJ/mol. The distribution and variation of the dependent variable is shown in Figure 7 – b) and it is similar to the one obtained for the training set (Figure 7 – a)). A complete table with NIST Web book/CRC ID, CASRN, compound name, SMILES and experimental values for the standard molar enthalpy of formation of gas phase at 298.15 K is provided in the *Additional file*4.

### Molecular descriptors

This research is based on the assumption that there is an underlying relationship between molecular structure and properties. Also, it is assumed that the multivariate molecular representation of the set of compounds reveals these analogies, i.e. physical and chemical properties of a chemical substance can be computed from its molecular structure, encoded in a numerical form with the aid of various descriptors. The key step in developing models is the selection of an informative and representative dataset. A total of 1485 molecular descriptors are used in this work and they were calculated using three main sources. The full descriptor set for each molecule of the training and independent validation sets is provided as supplementary material (*Additional files*3 and 4):

•***Molecular Descriptors generated by E-DRAGON***[55,56]***-*** E-DRAGON is the free online version of DRAGON and it generates a matrix of 1666 molecular descriptors for the dataset based on the compounds’ structure [13]. The 3D atomic coordinates of the lower energy conformation for the provided molecules were calculated using CORINA [57]. A preprocessing step was carried out and all zero variance variables (i.e. all the observations are the same) were removed, reducing the initial set to 1273 molecular descriptors. The high number of zero variance variables is due to the fact that this study deals only with hydrocarbons, therefore all descriptors related to other atoms than carbon and hydrogen have the value zero;

•***Simple Structural Descriptors*** - The calculation of eight specific molecular features (such as molecular weight, average molecular weight, number of ring(s), number of bonds in ring(s) and atom multiplicity (number of primary, secondary, tertiary and quaternary carbon atoms)) was performed using the molecular structure and the descriptors were added, one-by-one, based on a preliminary analysis of the results obtained with different combinations of descriptors;

•***Daylight Fingerprints***[50]**-** are binary hashed bit-strings of 1024 bits (FP2) representing fragments up to seven atoms, calculated using OpenBabel [58]. A preprocessing step was carried out and all zero variance variables were removed, reducing the initial set to 204 descriptors [58].

### Support vector machines

Support Vector Machines (SVMs) [48] are non-linear supervised learning methods for classification or prediction. SVMs construct a decision hyper plane or set of hyper planes in a high-dimensional feature space that minimizes the margin using a kernel function to transform the data, i.e., separate them based on the largest distance to the nearest training data points. This algorithm can optimize the function to a global optimum and the results have good predictive performance [59,60], being currently one of the most used methodologies for QSAR/QSPR studies. The disadvantage of SVMs is the lack of transparency of results due to its non-parametric nature and the sensitivity of the algorithm to the choice of kernel parameters. It produces good results and generalizes well even if the ratio between the number of variables and the number of observations becomes very unfavourable or in the presence of highly correlated predictors. Another advantage is the kernel-based system since it is possible to construct a non-linear model without explicitly having to produce new descriptors. The accuracy of an SVM model is dependent on the selection of the model parameters. An Epsilon-Support Vector Regression analysis using the Gaussian radial basis function (RBF) kernel (general-purpose kernel used when there is no prior knowledge about the data) has two parameters: cost (represents the penalty associated with large errors, increasing this value causes closer fitting to the training data) and gamma (controls the shape of the separating hyper plane, increasing this value usually increases the number of support vectors).

For the present study, the SVM implementation used was provided by the e1071 [61] package from R. This library provides an interface to libsvm which allows classification or regression [62,63]. Hyperparameter tuning in SVM models is done using the tune framework which is computationally expensive, considering that it performs a grid search over cost and gamma ranges.

### Random forests

Random Forests [46,64] are a non-linear consensus method for classification or regression that ensemble unpruned decision trees for a good generalization. In the decision tree the leaves represent the property/activities values and branches represent conjunctions of descriptors that lead to those properties/activities. Each tree is constructed independently of previous trees using a different bootstrap sample of data with replacement and where each node is split using the best subset of predictors randomly chosen at that node. The generalization of this method depends on the strength of the individual trees in the forest and the correlation between them. This algorithm only requires the selection of two parameters and it is usually not very sensitive to their values: the number of variables in the random subset at each node and the number of trees in the forest. In the end, new data is predicted by averaging the predictions made by all the trees in the forest. The algorithm Random Forest has several characteristics that make it suitable for QSAR/QSPR datasets [44-46]: a) it can be used when there are more variables than observations; b) it has a good predictive performance even when *noisy* variables are present; c) it is not very sensitive to the algorithm parameters, therefore there is a minimal necessity to tune the default parameters to achieve a good performance; d) due to its nature encompassing a large number of simple models, it largely reduces the problems caused by over fitting; e) it can handle a mixture of categorical and continuous descriptors; f) it returns measures of descriptor importance; g) there are high quality and free implementations of the method [44-46]. In random forests, there is no need for cross-validation or a separate test set to get an unbiased estimate of the test set error. It is estimated internally considering that each tree is constructed using a different bootstrap sample from the original data. About one-third of the cases are left out of the bootstrap sample (out of the bag (OOB) samples) and not used in the construction of the forest. These OOB samples are used to get a running unbiased estimate of the regression error as trees are added to the forest and they are also used to get estimates of variable importance. The proportion of variation explained indicates how well the set of molecular descriptors is able to explain the variation in the property/activity value.

The Random Forest implementation used in this work was provided by the R library randomForest [65].

### Variable importance

The ensemble voting procedure of random forests allows for the calculation of an importance score for each variable in the model. There are several available measures of variable importance. One of the most common measures is determined by looking at how much prediction error increases when the value of a variable in a node of a tree is permuted randomly while all others are left unchanged [43,45,46,64]. However, there is an issue in determining the variable importance of correlated variables, considering that in this determination it is assumed that each variable is independent of the response variable as well as from all other predictors [66]. Therefore, if correlated predictors are not independent, they obtain high importance scores and consequently, a higher probability of being selected for the split. Nevertheless, some correlated variables do hold predictive value, but only because of the truly important variable [66].

### Variable importance for feature selection

It is possible to use the variable rankings according to their importance in RFs models as a criterion for variable selection in other models [43,45]. The procedure followed in this work involved a sequence of steps in order to ensure coherence and results reproducibility. Therefore the procedure followed can be schematized with the following sequence of steps: (1) For the study problem, an initial systematic evaluation of the optimal model parameters was performed, and the results with the out-of-bag (OOB) root mean square error were evaluated for selecting the best possible parameter combination; (2) With the best parameter set, perform 10 model runs and record each variable importance score and rank, and using this new consensus ranking, define a sorted list starting with the most relevant variables and ending with the less important ones; (3) Proceed stepwise by feeding another prediction model (as an SVM) a progressively larger vector of input variables, following the ranked order. With such procedure it is expected that a minimal descriptor set, significantly smaller than the initial variable list may be found.

### Genetic algorithms

A genetic algorithm [67,68] is a meta-heuristic based on the application of a computational simplification of the biological evolutionary model over binary representations of solutions of a combinatorial optimization problem. Each solution is named a chromosome (or an individual), and its fitness is determined according to its result using an evaluation function. The algorithm starts by initiating a randomly generated set of solutions (named a population of chromosomes) and iteratively applies the evolutionary concepts of mutation, crossover and Darwinian selection to produce a new population. The process of selection is particularly important as an individual has a larger probability of being selected for the new generation according to its fitness, leading each generation to become progressively better than the original one. The meta-heuristic process is repeated for a given number of iterations.

Genetic algorithms have been used for feature selection problems in QSPR and QSAR studies [49,69,70]. For feature selection, generally a chromosome is modelled as a binary string identifying the selected features for a given prediction model. Typical models can be linear regression, Support Vector Machines or Neural Networks [69,71-73]. The evaluation function for each chromosome can then be a statistic of the application of the selected features using the predefined model to a validation set. Chromosomes with better validation results will tend to have a larger representation in the new population. The new population can then be changed using the cross over and mutation operators. Mutation changes randomly the solution by a fixed amount, causing some new features appear in the solution and others disappear, therefore guaranteeing that all available features will have a chance of being evaluated during a set of generations. Cross over, on the other hand, will allow the exchange of features selected between chromosomes within the same generation. After mutation and crossover the new population is evaluated again and the process is repeated for a number of iterations or until a suitable solution has been found.

A genetic algorithm was adapted to this problem and implemented considering the following parameters: *a)* the number of chromosomes – this parameter indicates how many solutions are being evolved simultaneously; *b)* the mutation rate – indicates the likelihood of a given feature is swapped into or out from a solution (chromosome) a value of 0.05 indicates that each feature has a probability of 5% of being changed. To avoid large drifts, the only mutation possibility is a swap, meaning that for each feature that leaves the solution, another one, not previously there, has to enter; *c)* the crossover rate – indicates how likely two chromosomes can exchange variables in the models; *d)* the solution density – indicates how many features can be selected for each solution; *e)* the selection factor – indicates the likelihood that a given solution can be selected for the new population and it is a function of its rank among the current population, better chromosomes mean that the respective solution or combination of features produces an improved model compared to the others. Superior models are ranked higher, and higher ranking models have an increasingly large probability of selection using a negative exponential distribution**.** Smaller values of the selection factor indicate a very small probability of selecting the worst solutions for the new generation, while larger values emphasize the possibility of selecting substandard solutions. All parameters were subject to a preliminary optimization process, so that it was possible that the implementation could explore a significant fraction of the solution space.

### Principal component analysis

Principal Component Analysis (PCA) [74] is a procedure based on the transformation of the variable space into linear orthogonal combinations that are ranked according to the explained variance of each combination (named a principal component). Thus, the first principal component is a linear combination of optimally-weighted observed variables that accounts for the maximal amount of total variance. The following components account for a maximal amount of variance in the observed variables that was not accounted for by the preceding components and they are linearly uncorrelated with all of the preceding components. PCA is fast to compute, easy to implement and several computer applications implement it [75]. This method guarantees to find a lower dimensional representation of the data on a linear subspace if such representation exists. PCA method can only identify gross variability as opposed to distinguishing among and within groups’ variability and the non linear combinations in the data cannot be efficiently exploited [24,76,77]. Principal components have been used as model inputs, when the variable space is too large and, specially, when models are particularly sensitive to the number of variables (e.g. Neural Networks) [78].

The procedure followed involved a sequence of steps in order to ensure coherence and reproducibility of results. This procedure can be schematized with the following sequence of steps: (1) The descriptor set in study was centered and scaled to mean equal to zero and standard deviation equal to one. (2) The PCA was used and the obtained principal components were organized in descending order of variance explained. (3) The PCs were used as a SVM model input following a stepwise procedure using the defined order. This method is aimed mainly to simplify the model fitting phase, as it does still require that all variables are computed.

### Model evaluation

The examination of the models’ fitness is performed through the comparison of the experimental and predicted properties and is needed to statistically ensure that the models are sound. The proportion of variation explained by the model and the root mean squared error (RMSE) are performed to determine the goodness of fit of the model. The explained variation measures the proportion to which a model accounts for the variance of the given data set. The concept of variation explained is, in many cases, equivalent to the correlation coefficient, however, for non linear models it is more adequate to present the explained variance [79]. Nevertheless, since in QSPR/QSAR studies it is standard to use the cross-validated squared correlation coefficient (q^2^), this terminology is adopted through the manuscript. In order to validate the robustness and predictive ability of the models, all results presented in this manuscript are the outcome of 10-fold cross validation or out-of-bag prediction. The process of cross-validation begins with the random division of the dataset into 10-folds of compounds. One partition is removed and used as test set and the model is created from the remaining data points, this process is repeated 10 times. The validation statistics are averaged over the rounds. An external validation with an independent dataset is considered optimal when evaluating how well the equation generalizes the data. The training set was used to derive a model that was further used to predict the properties of the test set instances, which were not used in the model development. The predictive proportion of variation explained (Q^2^) by the model and the root mean squared error (RMSE) are performed to determine the external predictive ability of the model.

## Competing interests

The authors declare that they have no competing interests.

## Authors’ contributions

ALT under the guidance and support of AOF designed the study, carried out the work flow and performed the analysis of the results. AOF designed and implemented the genetic algorithm. All authors contributed to the manuscript writing and approved its final version.

## Supplementary Material

Additional file 1 **List of descriptors selected using different selection/reduction methods: principal components analysis, genetic algorithms and variable importance calculated by random forests. **For principal components analysis, the list of variables and respective factor loadings are presented for the ten fist principal components (PC1 – PC10) , which are enough to explain 70.87% of the variance in the original dataset. For genetic algorithms, the number of times that each variable is selected in a total of 10 runs is presented. For variable importance calculated by random forests, a list of the variables is presented, along with their average and standard deviation of the importance score in the ten runs (ordered according to the average variable importance score). Click here for file

Additional file 2 **List of the 89 most important descriptors. **Table containing the 89 most important descriptors selected using variable importance calculated by random forests. The descriptors are presented in decreasing order of variable importance. Click here for file

Additional file 3 **Training Set. **Table containing information about the structure (ThermInfo ID, CASRN, compound name and SMILES) , the corresponding experimental values for the standard molar enthalpy of formation (kJ/mol) of gas phase at 298.15 K and the complete list of molecular descriptors for the compounds in the training set used in this study. More information about each compound can be found at http://therminfo.lasige.di.fc.ul.pt. Click here for file

Additional file 4 **Independent Validation Set. **Table containing information about the structure (NIST Web book/CRC ID, CASRN, compound name and SMILES), the corresponding experimental values for the standard molar enthalpy of formation (kJ/mol) of gas phase at 298.15 K and the complete list of molecular descriptors for the compounds in the independent validation set used in this study. More information about each compound can be found in the CRC Handbook of Chemistry and Physics or NIST Chemistry WebBook (http://webbook.nist.gov/chemistry/). Click here for file
